# Maternal and Infant Anthropometric Characteristics and Breast Cancer Incidence in the Daughter

**DOI:** 10.1038/s41598-020-59527-w

**Published:** 2020-02-13

**Authors:** Daniela Schmid, Walter C. Willett, Ming Ding, Karin B. Michels

**Affiliations:** 1 Institute for Prevention and Cancer Epidemiology, Faculty of Medicine and Medical Center, University of Freiburg, Freiburg, Germany; 2 Division for Quantitative Methods in Public Health and Health Services Research, Department of Public Health, Health Services Research, and Health Technology Assessment, UMIT – Private University for Health Sciences, Medical Informatics and Technology, Hall in Tyrol, Austria; 3 Department of Nutrition, Harvard T.H. Chan School of Public Health, Boston, MA USA; 4 Department of Epidemiology, Harvard T.H. Chan School of Public Health, Boston, MA USA; 5 Channing Division of Network Medicine, Brigham and Women’s Hospital and Harvard Medical School, Boston, MA USA; 6 Department of Epidemiology, Fielding School of Public Health, University of California, Los Angeles, CA USA

**Keywords:** Breast cancer, Risk factors

## Abstract

The intrauterine and early life environments have been linked to the etiology of breast cancer in prior studies. We prospectively examined whether maternal and newborn anthropometric factors as reported by the mother are related to an increased incidence of adult breast cancer in the daughter. We used data from 35,133 mother-daughter dyads of the Nurses’ Health Study (NHS) II and the Nurses’ Mothers’ Cohort Study. In 2001, living mothers of NHS II participants who were free of cancer completed a questionnaire on their pregnancy with the nurse and their nurse daughter’s early life experience. During 403,786 years of follow-up, 865 daughters developed incident cases of invasive breast cancer. Nurses with a birthweight of ≥4000 g had a 32% greater risk for breast cancer (multivariable-adjusted hazard ratio (HR) = 1.32, 95% confidence interval (CI) = 1.02–1.71, p-trend = 0.09) compared with those with birthweights of 3000–3499 g. Higher birth length tended to increase risk of premenopausal breast cancer (p for trend = 0.05). We further noted a modest U-shaped relation between maternal weight gain during pregnancy and premenopausal breast cancer incidence in the daughter. Fetal growth may contribute to shaping later life risk for breast cancer, especially prior to menopause.

## Introduction

Breast cancer is the most common malignancy among women worldwide, with an estimated 1.67 million new cancer cases diagnosed in 2012^1^. Known and suspected risk factors for breast cancer operating during adult life leave substantial variation in rates of this tumor unexplained. Over the past couple of decades perinatal and early life characteristics have emerged as novel breast cancer risk factors with consistency across different study populations but with some international divergence.

In prior epidemiologic studies, high birthweight has been associated with greater risk of breast cancer^2–6^, although this is not true for all studies^5,7^. Intrauterine exposure to sex steroid hormones, growth hormone, insulin, insulin-like growth factors (IGF)-1, and IGF-2, and epigenetic variation are potential key pathways linking anthropometric variables in early life to adult breast cancer risk^8^. Because investigations from retrospective case-control studies relying on information collected from the cases and controls themselves leave room for differential misclassification, data from prospective longitudinal analyses are warranted to overcome this bias. In previous literature, fewer data have been available on maternal factors such as pre-pregnancy body mass index (BMI), and weight gain during pregnancy in relation to the risk of breast cancer in the daughter. However, with the global epidemic of obesity maternal body mass prior to and during pregnancy has substantially increased during the past couple of decades, with 39% of women with normal, 59% with overweight, and 56% with obese prepregnancy BMI exceeding the current U.S. Institute of Medicine (IOM) recommendations for gestational weight gain^9^.The implications of these rapidly changing intrauterine conditions for the daughters’ future breast cancer risk need to be investigated.

We therefore used data from the prospective Nurses’ Mothers’ Cohort Study to explore the relation of anthropometric variables in newborns including birthweight and birth length to their risk of developing breast cancer in adulthood; we also examined whether maternal pre-pregnancy BMI, height, and weight gain during pregnancy as reported by the mothers were associated with the incidence of breast cancer among daughters participating in the Nurses’ Health Study (NHS) II.

## Methods

### Study population

The NHS II cohort was established in 1989 when 116,680 nurses aged between 25 and 42 years from 14 U.S. states completed a mailed questionnaire on lifestyle factors and medical history^10^. Follow-up questionnaires were mailed to participating nurses every two years updating information on lifestyle factors and health. In 2001, 35 830 living mothers of NHSII who were cancer-free participants completed a questionnaire on their pregnancy with their nurse daughter and on her early life exposures forming the Nurses’ Mothers’ Cohort Study^11^. NHS II participants who were adopted or whose adoption status was unknown and those with missing information on the exposures of interest were excluded from the respective analyses.

The institutional review boards of the Brigham and Women’s Hospital and Harvard School of Public Health, Boston approved the study protocol. Response to the questionnaire was considered implied consent.

### Exposure assessment

On the Mothers’ questionnaire, the nurses’ mothers were asked to report their height and weight before pregnancy in open-ended questions; this information was used to calculate pre-pregnancy BMI as kg/m^2^. Pre-pregnancy BMI was categorized following standard World Health Organization definitions of underweight (<18.5 kg/m^2^), normal weight (18.5–24.9 kg/m^2^), overweight (25–29.9 kg/m^2^), and obese (≥30 kg/m^2^). Mothers were also asked to report their gestational weight gain in pre-specified categories (<10, 10–14, 15–19, 20–29, 30–40, >40 lbs). Mothers further reported birthweight and birth length of their daughters as open-ended values. Birthweights recalled by the nurses’ mothers were highly concordant with birthweight information obtained from birth certificates (r = 0.85)^12^. Birthweight was coded using standard categories (<2500, 2500–2999, 3000–3499, 3500–3999, ≥4000 grams). Birth length was categorized in equally spaced intervals capturing the narrow distribution (<47, 47–49, 50–52, 53–55, >55 cm). Ponderal index was calculated from birth weight and length [Ponderal index = birthweight (grams) x 100/(birth length, cm)³] and categorized in quintiles.

### Covariate information

Covariate information on tobacco use during pregnancy and gestational age was obtained from the Mothers’ questionnaire. Additional information on daughter’s characteristics, including race/ethnicity, family history of breast cancer, month and year of birth of the nurse, menopausal status, adult caloric intake, alcohol consumption, smoking, and BMI was available from the NHS II questionnaires. The derivation of menopausal status in this cohort has been previously described^13^.

### Ascertainment of breast cancer

On follow-up questionnaires administered every two years, NHS participants were asked whether they had been diagnosed with breast cancer in the previous two years. To confirm the diagnosis, participants who reported a breast cancer diagnosis were asked for permission to review their relevant medical records and pathology reports. Due to the high accuracy of self-reported breast cancer diagnosis (>98%), nurse participants who reported a diagnosis of breast cancer but for whom medical records could not be obtained were classified as breast cancer cases.

### Ascertainment of deaths

Deaths were identified by reports from the next of kin, postal authorities, or the National Death Index.

### Statistical analysis

Cox proportional hazards regression models were used to estimate the hazard ratios (HRs) and 95% confidence intervals (CIs) of the incidence of breast cancer in the daughters across categories of infant birthweight, birth length, maternal pre-pregnancy BMI, height, and gestational weight gain. Participants contributed follow-up time from the return of the Nurses’ Mothers questionnaire in 2001 to the date of death, cancer diagnosis (except non-melanoma skin cancer), or end of follow-up on June 1, 2013. The basic model adjusted for the nurse’s age and follow-up cycle. Multivariable-adjusted models additionally included race (White, Non-White), family history of breast cancer (yes, no), maternal pre-pregnancy BMI (<18.5 kg/m^2^, 18.5–24.9 kg/m^2^, 25.0–30.0 kg/m^2^, >30 kg/m^2^), pregnancy weight gain (<10 lbs, 10–14 lbs, 15–19 lbs, 20–29, 30–40, +40 lbs), maternal smoking during pregnancy (no smoking, quit smoking during pregnancy, light smoker [<15 cigarettes/day], heavy smoker [≥15 cigarettes/day]), and gestational age (<38 weeks, 38–42 weeks, >42 weeks). In separate models, we included adult characteristics of the nurses including alcohol consumption, energy intake, smoking habits, and most recent pre-diagnostic (current) BMI during adulthood, all of which were updated during follow-up.

We further stratified our analytic models by menopausal status. To test for a linear trend across categories of exposures, we created a continuous variable using the midpoint of each category. Statistical tests were considered statistically significant at the 5% level. All statistical analyses were performed using the SAS statistical software version 9.4 (SAS Institute Inc, Cary, NC). The study has been conducted and reported in accordance with the Strengthening the Reporting of Observational Studies in Epidemiology (STROBE) guidelines^14^.

## Results

During 403,786 person-years of follow-up between 2001 and 2013, we documented 865 newly diagnosed invasive cases of breast cancer among NHS II participants. The distribution of perinatal and adult characteristics of the nurse daughters and of the maternal anthropometric pregnancy characteristics across categories of birthweight and pregnancy weight gain is shown in Tables 1 and 2, respectively. Birthweight was correlated with birth length and gestational age. Mothers of daughters with greater birthweight were more likely to have smoked less than those with a low birthweight, to have gained more weight during pregnancy, and had a slightly higher pre-pregnancy BMI. Mothers who smoked during pregnancy gained the least amount of weight on average.

**Table 1:** Age-standardized characteristics of NHS II participants and their mothers participating in the Nurses’ Mothers’ Cohort Study according to the daughters’ birthweight.

	Nurse’s birthweight				
	<2500 g	2500–2999 g	3000–3499 g	3500–3999 g	≥4000 g
***Nurse’s characteristics***					
Age at baseline, years	51.3 (5.8)	51.1 (5.8)	50.9 (5.7)	50.8 (5.7)	50.6 (5.8)
Race, white, %	94.9	96.2	96.6	97.3	97.3
Family history of breast cancer, %	15.4	15.0	14.5	14.9	14.5
Birth length, cm	46.5 (4.2)	48.8 (3.4)	50.7 (3.3)	52.1 (3.1)	53.6 (3.0)
Adult smoking, %	6.2	6.7	6.1	6.0	5.9
Adult alcohol intake, g/d	1.8 (1.6)	1.9 (1.6)	1.9 (1.6)	1.9 (1.6)	1.8 (1.6)
Adult energy intake, kcal/d	1805.5 (557.5)	1818.4 (556.2)	1825.4 (554.2)	1836.9 (553.5)	1834.2 (567.5)
Adult BMI, kg/m²	27.1 (6.2)	26.7 (6.1)	26.7 (6.1)	27.3 (6.4)	28.0 (6.8)
***Mother’s characteristics***					
Maternal height, m	162.3 (6.5)	162.4 (6.2)	163.3 (6.2)	164.4 (6.3)	165.3 (6.2)
Pre-pregnancy BMI, kg/m²	20.8 (2.7)	20.9 (2.5)	21.2 (2.5)	21.6 (2.8)	22.1 (3.0)
Smoking during pregnancy, %	34.1	28.0	20.7	14.9	10.8
Pregnancy weight gain, lbs	20.3 (8.4)	21.9 (7.9)	23.5 (8.0)	25.3 (8.0)	27.3 (8.4)
Gestational age, wks	36.8 (3.4)	39.0 (2.1)	39.6 (2.0)	39.9 (2.1)	40.2 (2.2)

BMI = body mass index; values are means (SD) or percentages and are standardized to the age distribution of the study population (except age).

**Table 2:** Age-standardized characteristics of NHS II participants and their mothers participating in the Nurses’ Mothers’ Cohort Study according to the mothers’ weight gain during pregnancy.

	Weight gain during pregnancy (lbs)					
	<10	10–14	15–19	20–29	30–40	40+
***Nurse’s characteristics***						
Age at baseline, years	51.1 (5.9)	51.27 (5.8)	50.9 (5.7)	50.7 (5.7)	50.9 (5.7)	51.0 (5.8)
Race, white, %	95.0	97.0	96.6	97.0	97.1	96.1
Family history of breast cancer, %	15.8	14.6	14.9	14.8	14.3	13.6
Birth length, cm	49.7 (4.4)	49.8 (4.1)	50.3 (3.7)	50.7 (3.5)	51.2 (3.6)	51.3 (3.6)
Adult smoking, %	6.0	6.8	6.0	5.9	6.7	7.3
Adult alcohol intake, g/d	1.7 (1.6)	1.9 (1.6)	1.9 (1.6)	1.9 (1.6)	1.9 (1.6)	1.9 (1.6)
Adult energy intake, kcal/d	1787.1 (554.3)	1825.8 (549.2)	1832.8 (551.9)	1829.7 (557.6)	1822.7 (555.7)	1819.6 (556.7)
Adult BMI, kg/m²	28.2 (7.1)	27.0 (6.2)	26.6 (6.0)	26.7 (6.1)	27.5 (6.5)	28.1 (6.7)
***Mother’s characteristics***						
Height	163.5 (6.8)	163.1 (6.6)	163.2 (6.3)	163.6 (6.2)	163.8 (6.2)	164.2 (6.4)
Pre-pregnancy BMI, kg/m²	22.4 (3.9)	21.1 (2.7)	21.1 (2.5)	21.3 (2.4)	21.4 (2.7)	20.9 (2.9)
Smoking during pregnancy, %	25.3	21.8	21.1	20.9	21.1	21.8
Gestational age, wks	39.0 (2.8)	39.08 (2.55)	39.3 (2.3)	39.5 (2.2)	39.6 (2.2)	39.8 (2.4)

BMI = body mass index; values are means (SD) or percentages and are standardized to the age distribution of the study population (except age).

Compared with daughters with birthweights of 3000–3499 g, those with a birthweight of ≥4000 g had a 32% greater incidence of adult breast cancer (multivariable-adjusted HR = 1.32, 95% CI = 1.01–1.71, p for trend: 0.10; Table 3). This association was restricted to premenopausal breast cancer (multivariable-adjusted HR = 1.32, 95% CI = 0.89–1.96), while no association was observed for postmenopausal breast cancer (multivariable-adjusted HR = 1.05, 95% CI = 0.69–1.59). We further noted a statistical trend of borderline significance for a greater risk of premenopausal breast cancer in daughters with increasing birth length (p for trend = 0.06; Table 4). Due to the high correlation between birthweight and birth length (0.47) we chose not to mutually adjust the analyses of these two variables. We also explored the role of Ponderal Index but did not observe any association with adult breast cancer incidence (Tables 3 and 4).

**Table 3:** HRs (and 95% CIs) for invasive breast cancer incidence between 2001 and 2013 by categories of anthropometric characteristics at birth among NHS II participants whose mothers participated in the Nurses’ Mothers’ Cohort Study.

	All Women			
	N cases/person-yrs	Age-adjusted	Multivariable-adjusted model 1	Multivariable- + adult variables adjusted model 2
***Birthweight***				
<2500g	53/25444	0.97 (0.72–1.29)	0.92 (0.67–1.25)	0.92 (0.67–1.25)
2500–2999 g	155/77004	0.92 (0.76–1.12)	0.91 (0.75–1.10)	0.91 (0.75–1.10)
3000–3499 g	343/160285	1.00	1.00	1.00
3500–3999 g	208/100081	0.99 (0.83–1.17)	0.98 (0.83–1.17)	0.98 (0.83–1.17)
≥4000g	72/26626	1.30 (1.01–1.68)	1.32 (1.01–1.71)	1.32 (1.02–1.71)
*P* trend		0.13	0.10	0.09
***Birth length***				
<47 cm	78/34518	1.05 (0.80–1.39)	1.06 (0.80–1.40)	1.05 (0.80–1.39)
47–49 cm	144/82314	0.81 (0.64–1.02)	0.81 (0.64–1.02)	0.81 (0.64–1.02)
50–52 cm	151/71922	1.00	1.00	1.00
53–55 cm	197/87713	1.07 (0.87–1.33)	1.07 (0.87–1.33)	1.07 (0.87–1.33)
>55 cm	38/18543	0.97 (0.68–1.39)	0.98 (0.68–1.40)	0.98 (0.68–1.40)
*P* trend		0.14	0.15	0.14
***Ponderal index***				
Quintile 1	111/58733	0.92 (0.70, 1.19)	0.93 (0.71, 1.21)	0.92 (0.71, 1.20)
Quintile 2	143/58682	1.24 (0.97, 1.59)	1.24 (0.96, 1.59)	1.23 (0.96, 1.58)
Quintile 3	115/57510	1.00	1.00	1.00
Quintile 4	121/59631	1.01 (0.78, 1.31)	1.02 (0.78, 1.32)	1.02 (0.78, 1.32)
Quintile 5	118/58633	0.99 (0.76, 1.28)	0.98 (0.75, 1.27)	0.97 (0.75, 1.27)
*P* trend		0.79	0.71	0.73

Ponderal index = birthweight (g) x 100/(birth length, cm)³]

HR = hazard ratio, CI = confidence interval; Age-adjusted models adjusted for age of nurse (continuous).

Multivariable-adjusted model 1 includes age of nurse (continuous), race (White, Non-White), family history of breast cancer (yes, no), pre-pregnancy body mass index (<18.5 kg/m^2^, 18.5–24.9. kg/m^2^, 25.0–30.0 kg/m^2^, >30 kg/m^2^), weight gain during pregnancy (<10 lbs, 10–14 lbs,15–19 lbs, 20–29 lbs, 30–40 lbs, ≥40 lbs), smoking during pregnancy (no smoking, quit smoking during pregnancy, light smoker (<15 cigarettes/day), heavy smoker (≥15 cigarettes/day), and gestational age (<38 weeks, 38–42 weeks, >42 weeks).

Multivariable-adjusted model 2: same as model 1 but with additional adjustment for adult caloric intake (quintiles), adult alcohol intake (quintiles), adult smoking (never, past, current <15 cigarettes/d, current ≥15 cigarettes/d), and adult body mass index (<20.9 kg/m^2^, 21–22.9 kg/m^2^, 23–24.9 kg/m^2^, 25–29.9 kg/m^2^, 30–34.9 kg/m^2^, +35 kg/m^2^).

**Table 4:** HRs (and 95% CIs) for pre- and postmenopausal breast cancer incidence between 2001 and 2013 by categories of anthropometric characteristics at birth among NHS II participants whose mothers participated in the Nurses’ Mothers’ Cohort Study.

	Premenopausal Women				Postmenopausal Women			
	N cases/person-yrs	Age-adjusted	Multivariable-adjusted model 1	Multivariable- + adult variables adjusted model 2	N cases/person-yrs	Age-adjusted	Multivariable-adjusted model 1	Multivariable- + adult variables adjusted model 2
***Birthweight***								
<2500g	23/11025	1.06 (0.67–1.67)	0.93 (0.57–1.52)	0.91 (0.56–1.49)	27/12025	0.98 (0.65–1.48)	1.05 (0.67–1.62)	1.04 (0.67–1.61)
2500–2999 g	54/34277	0.86 (0.62–1.18)	0.84 (0.60–1.16)	0.83 (0.60–1.15)	79/35004	0.92 (0.70–1.21)	0.92 (0.70–1.21)	0.92 (0.70–1.21)
3000–3499 g	139/73840	1.00	1.00	1.00	169/70930	1.00	1.00	1.00
3500–3999 g	90/47486	1.04 (0.79–1.36)	1.04 (0.79–1.36)	1.04 (0.79–1.36)	94/42592	0.94 (0.73–1.22)	0.92 (0.71–1.19)	0.93 (0.72–1.20)
≥4000g	33/13046	1.33 (0.90–1.95)	1.32 (0.89–1.96)	1.32 (0.89–1.96)	27/10885	1.05 (0.70–1.58)	1.05 (0.69–1.59)	1.07 (0.70–1.62)
*P* trend		0.16	0.10	0.09		0.81	0.97	0.97
***Birth length***								
<47 cm	26/15613	0.94 (0.59–1.49)	0.87 (0.54–1.39)	0.87 (0.54–1.39)	44/15365	1.29 (0.88–1.90)	1.39 (0.94–2.05)	1.38 (0.93–2.05)
47–49 cm	67/39088	0.94 (0.66–1.33)	0.91 (0.64–1.30)	0.90 (0.63–1.29)	56/35020	0.70 (0.48–1.00)	0.72 (0.50–1.04)	0.71 (0.50–1.03)
50–52 cm	60/35245	1.00	1.00	1.00	66/29794	1.00	1.00	1.00
53–55 cm	93/42464	1.22 (0.87–1.69)	1.21 (0.87–1.69)	1.22 (0.87–1.69)	85/36514	1.09 (0.79-1.51)	1.11 (0.80–1.53)	1.11 (0.80–1.54)
>55 cm	17/8523	1.12 (0.65–1.94)	1.13 (0.65–1.96)	1.14 (0.66–1.97)	19/8061	1.07 (0.64–1.79)	1.12 (0.66–1.87)	1.12 (0.67–1.88)
*P* trend		0.10	0.06	0.05		0.36	0.48	0.43
***Ponderal index***								
Quintile 1	44/26672	0.84 (0.56, 1.27)	0.86 (0.57, 1.30)	0.85 (0.56, 1.28)	55/26181	1.04 (0.70, 1.55)	1.09 (0.73, 1.63)	1.11 (0.75, 1.66)
Quintile 2	69/28758	1.26 (0.87, 1.81)	1.30 (0.90, 1.88)	1.28 (0.88, 1.85)	58/24287	1.24 (0.84, 1.84)	1.21 (0.81, 1.80)	1.22 (0.82, 1.81)
Quintile 3	54/28777	1.00	1.00	1.00	47/23179	1.00	1.00	1.00
Quintile 4	51/29014	0.90 (0.61, 1.32)	0.92 (0.62, 1.37)	0.91 (0.61, 1.34)	55/24624	1.11 (0.75, 1.65)	1.12 (0.75, 1.67)	1.15 (0.77, 1.72)
Quintile 5	45/27020	0.84 (0.56, 1.26)	0.84 (0.56, 1.26)	0.82 (0.54, 1.24)	55/25539	1.07 (0.72, 1.58)	1.05 (0.70, 1.56)	1.06 (0.71, 1.58)
		0.38	0.33	0.32		0.88	0.71	0.71

Ponderal index = birthweight (g) x 100/(birth length, cm)³].

HR = hazard ratio, CI = confidence interval.

Age-adjusted models adjusted for age of nurse (continuous). Multivariable-adjusted model 1 includes age of nurse (continuous), race (White, Non-White), family history of breast cancer (yes, no), pre-pregnancy body mass index (<18.5 kg/m^2^, 18.5–24.9. kg/m^2^, 25.0–30.0 kg/m^2^, >30 kg/m^2^), weight gain during pregnancy (<10 lbs, 10–14 lbs,15–19 lbs, 20–29 lbs, 30–40 lbs, ≥40 lbs), smoking during pregnancy (no smoking, quit smoking during pregnancy, light smoker (<15 cigarettes/day), heavy smoker (≥15 cigarettes/day), and gestational age (<38 weeks, 38–42 weeks, >42 weeks).

Multivariable-adjusted model 2: same as model 1 but with additional adjustment for adult caloric intake (quintiles), adult alcohol intake (quintiles), adult smoking (never, past, current <15 cigarettes/d, current ≥15 cigarettes/d), and adult body mass index (<20.9 kg/m^2^, 21–22.9 kg/m^2^, 23–24.9 kg/m^2^, 25–29.9 kg/m^2^, 30–34.9 kg/m^2^, +35 kg/m^2^).

We did not note any important overall associations between maternal height, pre-pregnancy BMI, or maternal weight gain during pregnancy and daughter’s breast cancer risk (Tables 5 and 6). Daughters whose mothers had a pre-pregnancy BMI of ≥25 kg/m^2^ experienced a multivariable-adjusted HR of breast cancer of 0.98 (95% CI: 0.74–1.30) compared to those whose mothers were normal-weight (BMI 18.5–24.9 kg/m^2^; Table 5). We noted a modest U–shaped relation of maternal weight gain during pregnancy with the risk of premenopausal breast cancer in the daughters, but not for postmenopausal breast cancer (Table 6). For premenopausal breast cancer incidence in daughters comparing low (<15 lbs) versus normal maternal weight gain (20–29 lbs) during pregnancy the multivariable-adjusted HR was 1.23 (95% CI = 0.88–1.71) and comparing high (40+ lbs) versus normal weight gain the multivariable-adjusted HR was 1.17 (95% CI = 0.70–1.95). Additional adjustment for variables of the nurse’s adult life did not appreciably modify the results.

**Table 5:** HRs (and 95% CIs) for invasive breast cancer incidence between 2001 and 2013 by categories of maternal height, pre-pregnancy BMI, and weight gain during pregnancy among NHS II participants whose mothers participated in the Nurses’ Mothers’ Cohort Study.

	All Women			
	N cases/person-yrs	Age-adjusted	Multivariable-adjusted model 1	Multivariable- + adult variables adjusted model 2
***Maternal height***				
<155 cm	85/40560	0.91 (0.72–1.16)	0.92 (0.72–1.16)	0.91 (0.72–1.16)
155–164.9 cm	368/176952	0.92 (0.80–1.06)	0.92 (0.80–1.06)	0.92 (0.79–1.06)
165–174.9 cm	380/170167	1.00	1.00	1.00
≥175 cm	32/16109	0.92 (0.64–1.32)	0.92 (0.64–1.33)	0.92 (0.64–1.32)
*P* trend		0.32	0.34	0.32
***Pre***–***pregnancy BMI***				
<18.5 kg/m^2^	95/40651	1.08 (0.87–1.34)	1.07 (0.86–1.32)	1.06 (0.85–1.32)
18.5–24.9 kg/m^2^	655/308860	1.00	1.00	
≥25 kg/m^2^	55/27229	0.98 (0.74–1.30)	0.98 (0.74–1.30)	1.02 (0.77–1.34)
*P* trend		0.48	0.56	0.67
***Maternal weight gain during pregnancy***				
Weight gain <15 lbs	118/54954	1.03 (0.83–1.27)	1.02 (0.82–1.26)	1.02 (0.83–1.27)
Weight gain 15–19 lbs	154/78127	0.96 (0.79–1.16)	0.96 (0.79–1.16)	0.97 (0.80–1.17)
Weight gain 20–29 lbs	320/156964	1.00	1.00	1.00
Weight gain 30–40 lbs	152/64455	1.16 (0.95–1.41)	1.16 (0.95–1.41)	1.16 (0.96–1.41)
Weight gain ≥40 lbs	47/20204	1.12 (0.82–1.53)	1.11 (0.82–1.52)	1.14 (0.83–1.55)
*P* trend		0.15	0.15	0.14

HR = hazard ratio, CI = confidence interval, BMI = body mass index.

Age-adjusted models adjusted for age of nurse (continuous).

Multivariable-adjusted model 1 includes age of nurse (continuous), race (White, Non-White), family history of breast cancer (yes, no),

smoking during pregnancy (no smoking, quit smoking during pregnancy, light smoker (<15 cigarettes/day), and heavy smoker (≥15 cigarettes/day), and models for pre-pregnancy BM and weight gain during pregnancy are mutually adjusted for weight gain during pregnancy (<10 lbs, 10–14 lbs,15–19 lbs, 20–29 lbs, 30–40 lbs, ≥40 lbs) and pre-pregnancy BMI (<18.5 kg/m^2^, 18.5–24.9. kg/m^2^, 25.0–30.0 kg/m^2^, >30 kg/m^2^).

Multivariable-adjusted model 2: same as model 1 but with additional adjustment for adult caloric intake (quintiles), adult alcohol intake (quintiles), adult smoking (never, past, current <15 cigarettes/d, current ≥15 cigarettes/d), and adult BMI (<20.9 kg/m^2^, 21–22.9 kg/m^2^, 23–24.9 kg/m^2^, 25–29.9 kg/m^2^, 30–34.9 kg/m^2^, +35 kg/m^2^).

**Table 6:** HRs (and 95% CIs) for pre- and postmenopausal breast cancer incidence between 2001 and 2013 by categories of maternal height, pre-pregnancy BMI, and weight gain during pregnancy among NHS II participants whose mothers participated in the Nurses’ Mothers’ Cohort Study.

	Premenopausal Women				Postmenopausal Women			
	N cases/person-yrs	Age-adjusted	Multivariable-adjusted model 1	Multivariable- + adult variables adjusted model 2	N cases/person-yrs	Age-adjusted	Multivariable-adjusted model 1	Multivariable- + adult variables adjusted model 2
***Maternal height***								
<155 cm	33/17643	0.91 (0.62–1.34)	0.92 (0.63–1.35)	0.90 (0.61–1.32)	40/18739	0.87 (0.62–1.23)	0.87 (0.62–1.24)	0.87 (0.61–1.23)
155–164.9 cm	142/80230	0.90 (0.71–1.13)	0.91 (0.72–1.14)	0.90 (0.71–1.13)	189/79574	0.96 (0.78–1.19)	0.97 (0.78–1.19)	0.96 (0.78–1.18)
165–174.9 cm	156/79486	1.00	1.00	1.00	178/73777	1.00	1.00	1.00
≥175 cm	14/7883	0.86 (0.49–1.53)	0.86 (0.49–1.52)	0.87 (0.49–1.53)	14/6628	0.92 (0.53–1.58)	0.90 (0.52–1.56)	0.91 (0.52–1.57)
*P* trend		0.53	0.56	0.47		0.53	0.55	0.52
***Pre***–***pregnancy BMI***								
<18.5 kg/m^2^	33/17551	1.04 (0.72–1.50)	1.00 (0.69–1.45)	0.98 (0.68–1.43)	44/18993	0.97 (0.70–1.33)	0.97 (0.70–1.33)	0.97 (0.70–1.33)
18.5–24.9 kg/m^2^	266/142445	1.00	1.00		321/135966	1.00	1.00	1.00
≥25 kg/m^2^	24/13426	0.89 (0.58–1.38)	0.86 (0.56–1.33)	0.88 (0.57–1.36)	27/11010	1.10 (0.74–1.63)	1.11 (0.75–1.66)	1.14 (0.76–1.70)
*P* trend		0.65	0.70	0.80		0.68	0.65	0.62
***Maternal weight gain during pregnancy***								
Weight gain <15 lbs	51/24064	1.23 (0.88–1.70)	1.23 (0.88–1.71)	1.24 (0.89–1.72)	50/25453	0.80 (0.58–1.10)	0.78 (0.57–1.08)	0.78 (0.57–1.08)
Weight gain 15–19 lbs	56/35940	0.94 (0.69–1.29)	0.94 (0.69–1.29)	0.95 (0.69–1.30)	77/34477	0.92 (0.70–1.21)	0.92 (0.70–1.21)	0.93 (0.70–1.22)
Weight gain 20–29 lbs	132/75463	1.00	1.00	1.00	159/66229	1.00	1.00	1.00
Weight gain 30–40 lbs	66/29900	1.25 (0.92–1.69)	1.26 (0.93–1.70)	1.28 (0.94–1.73)	70/27878	1.05 (0.79–1.40)	1.04 (0.78–1.39)	1.04 (0.78–1.38)
Weight gain ≥40 lbs	18/8872	1.21 (0.73–2.01)	1.17 (0.70–1.95)	1.17 (0.70–1.96)	24/9251	1.05 (0.68–1.62)	1.04 (0.67–1.61)	1.03 (0.67–1.60)
*P* trend		0.46	0.49	0.47		0.13	0.12	0.14

HR = hazard ratio, CI = confidence interval, BMI = body mass index.

Age-adjusted models adjusted for age of nurse (continuous).

Multivariable-adjusted model 1 includes age of nurse (continuous), race (White, Non-White), family history of breast cancer (yes, no),

smoking during pregnancy (no smoking, quit smoking during pregnancy, light smoker (<15 cigarettes/day), and heavy smoker (≥15 cigarettes/day), and models for pre-pregnancy BM and weight gain during pregnancy are mutually adjusted for weight gain during pregnancy (<10 lbs, 10–14 lbs,15–19 lbs, 20–29 lbs, 30–40 lbs, ≥40 lbs) and pre-pregnancy BMI (<18.5 kg/m^2^, 18.5–24.9. kg/m^2^, 25.0–30.0 kg/m^2^, >30 kg/m^2^).

Multivariable-adjusted model 2: same as model 1 but with additional adjustment for adult caloric intake (quintiles), adult alcohol intake (quintiles), adult smoking (never, past, current <15 cigarettes/d, current ≥15 cigarettes/d), and adult BMI (<20.9 kg/m^2^, 21–22.9 kg/m^2^, 23–24.9 kg/m^2^, 25–29.9 kg/m^2^, 30–34.9 kg/m^2^, +35 kg/m^2^).

## Discussion

In this study, a high birthweight was associated with a greater incidence of breast cancer later in life. Moreover, a positive trend with increasing birth length was noted for premenopausal breast cancer. We further observed a U-shaped relation between maternal weight gain during pregnancy and premenopausal breast cancer in daughters.

A positive association between high birthweight studies (from measurements made at birth by medical doctors, birth records/medical registers, maternal interviews, or adult self-report) and an increased risk of breast cancer in adulthood has been observed in most prior studies^2–8^ and confirms our earlier findings from the same cohort using self-reported birth weight^4,8^. The mechanisms underlying the association between a high birthweight and the diagnosis of breast cancer risk in later life is not entirely clear, but may be orchestrated by intrauterine exposures to growth hormones and epigenetic programming^8,15^. Birthweight is likely a marker for intrauterine levels of sex estrogen and progesterone, growth hormone, IGF-1, IGF-2, and insulin itself which may increase the number of susceptible stem cells in the mammary gland or enhance cell proliferation, thereby contributing to tumor development through accumulation of DNA mutations^8^. Since the epigenetic profile is established *in utero*, intrauterine stressors may affect the epigenetic pattern and therefor susceptibility to cancer^16^. The exposure to high levels of hormones and growth factors in utero may change the response of breast tissue to hormonal inputs later.

Our study revealed a statistically significant dose-response relation between birth length and premenopausal breast cancer. A previous meta-analysis suggested a positive association between birth length and later breast cancer in studies based on birth records, but not in studies based on parental recall or self-reports, likely reflecting the difficulty in precisely capturing birth length^7^. Unlike in our study, the birth length – breast cancer association was not modified by menopausal status in that meta-analysis^7^. The mechanisms underlying any association between birth length and subsequent breast cancer are likely similar to those connecting birthweight and breast cancer.

We observed a modest U-shaped relation between maternal weight gain during pregnancy and the daughter’s premenopausal breast cancer risk. A previous analysis by Sanderson *et al*.^17^ examining the relation between maternal weight gain during pregnancy reported by the mother and breast cancer risk in the daughters in two population-based case-control studies reported a higher breast cancer risk in daughters (HR = 1.50, 95% CI: 1.10–2.00) whose mothers gained 25–34 pounds compared to mothers who gained 15–24 pounds^17^. Notably, women whose mothers gained 35 pounds or more during pregnancy were not at an increased risk of breast cancer in that study^17^. We previously analyzed the relation between maternally reported pre-pregnancy BMI and gestational weight gain with breast cancer risk in adult daughters in a retrospective case-control study nested within the NHS I and NHS II and found no association^18^. Similar to the results in our current study, Sanderson *et al*.^17^ did not observe a link between maternal pre-pregnancy BMI and breast cancer risk in the daughters. However, both our study and that by Sanderson and colleagues were based on pregnancies several decades in the past, when in particular pre-pregnancy BMI, but also gestational weight gain recommendations were considerably lower than currently. Hence, these associations need to be further evaluated in more recent studies including a larger proportion of mothers with high pre-pregnancy BMI and maternal weight gain, reflecting current distributions.

Strengths of our study include its prospective design, the large number of study participants, and adjustment for a number of important potential perinatal confounding variables. Moreover, our study was based on a two-generation design providing the unique opportunity to use data collected directly from the mothers and combine them with data provided by the nurses.

A limitation of our study is that gestational and newborn characteristics had to be recalled by the mothers likely introducing random misclassification. However, the validity of birthweight reported by the mothers appears to be high when compared with birthweight recorded on state birth records (r = 0.85)^12^. Similarly, other studies have reported good agreement between pregnancy-related factors recorded during pregnancy and maternal recall^19–21^. The nurses’ adult life variables did not appreciably affect associations observed. However, we cannot rule out unmeasured confounding or residual confounding through covariates measured with error. Finally, with mothers being born between 1921 and 1964, we had limited power to examine substantial pregnancy weight gains and high values of pre-pregnancy BMI which have become more prevalent in recent years.

In summary, a high birthweight and possibly a high birth length may predict an increased risk of breast cancer in later life, particularly of premenopausal breast cancer. Whether maternal factors during pregnancy and other infant factors are related to adult breast cancer risk requires further clarification in other prospective studies with good exposure validity.

## Data Availability

All relevant data are provided within the manuscript or are available from the corresponding author upon request.
